# The value of the platelet/high-density lipoprotein cholesterol ratio in predicting depression and its cardiovascular disease mortality: a population-based observational study

**DOI:** 10.3389/fendo.2024.1402336

**Published:** 2024-07-29

**Authors:** Huifeng Zhang, Ying Xu, Yaying Xu

**Affiliations:** ^1^ Department of Cardiovascular, The First Affiliated Hospital, and College of Clinical Medicine of Henan University of Science and Technology, Luoyang, China; ^2^ Department of Hematology, The First Affiliated Hospital, and College of Clinical Medicine of Henan University of Science and Technology, Luoyang, China; ^3^ Department of Endocrinology, The First Affiliated Hospital, and College of Clinical Medicine of Henan University of Science and Technology, Luoyang, China

**Keywords:** high-density lipoprotein cholesterol, platelet/high-density lipoprotein cholesterol ratio, depression, NHANES, CVD mortality

## Abstract

**Background:**

Cardiovascular disease (CVD) and depression have a bidirectional association, with inflammation and metabolic factors being common important triggers for both conditions. However, as a novel inflammatory and metabolic marker, platelet-to-HDL-C ratio (PHR) has not been established in relation to depression and cardiovascular disease.

**Materials and methods:**

Participants aged 20 years and older were included in the 2005–2018 NHANES database. PHR was calculated as the ratio of platelet count (1000 cells/μL) to HDL-C (mmol/L). The Patient Health Questionnaire (PHQ-9) was used to diagnose depression, with a cutoff value of 10. Weighted logistic regression analysis and restricted cubic spline (RCS) analysis were employed to examine the association between PHR and depression-related features. Additionally, weighted COX regression and RCS were used to analyze the association of PHR with CVD mortality in patients with depression. Receiver operating characteristic curves were used to assess whether PHR had an advantage over HDL-C in predicting depression. Finally, the mediating role of PHR in the latest cardiovascular health indicator Life’s Essential 8 and depression was explored.

**Results:**

A total of 26,970 eligible participants were included, including 2,308 individuals with depression, representing approximately 160 million U.S. adults when weighted. After full adjustment, we estimated that the odds ratio (OR) of depression associated with a per standard deviation (SD) increase in PHR was 1.06 (95% CI: 1.01–1.12, P=0.03). The restricted cubic spline (RCS) analysis indicated a linear association (Nonlinear P=0.113). When PHR was divided into four groups based on quartiles and included in the model after full adjustment for depression risk factors, participants in quartile 2, quartile 3, and quartile 4 of PHR showed a trend of increasing risk of depression compared to the lowest quartile group (P trend=0.01). In addition, weighted COX regression and RCS revealed that a per SD increase in PHR was associated with a higher risk of CVD mortality among patients with depression (HR: 1.38, 95% CI: 1.05–1.81, P=0.02, Nonlinear P=0.400). Subgroup analyses showed that current alcohol consumption enhanced the association between PHR and depression (P for interaction=0.017). Furthermore, the areas under the ROC curves (AUC) were 0.556 (95% CI, 0.544–0.568; P < 0.001) for PHR and 0.536 (95% CI, 0.524–0.549; P < 0.001) for HDL-C (P_DeLong_ = 0.025). Finally, mediation analysis indicated that PHR was an intermediate mechanism between LE8 and depression (mediation proportion=5.02%, P=0.02).

**Conclusion:**

In U.S. adults, an increase in PHR linearly increases the risk of depression and CVD mortality among individuals with depression. Additionally, PHR has a better predictive advantage for depression compared to HDL-C. Furthermore, PHR significantly mediates the association between LE8 scores and depression.

## 1 Introduction

Depression is one of the most common mental disorders and is considered the most significant type among modern psychological disorders (1). Currently, approximately 1 in every 20 individuals is at risk of developing depression, and it is projected to become the leading burden of disease globally within the next five years (1, 2). In the United States, the prevalence of depression is even higher among adults, with approximately 1 in every 7 individuals affected by this condition (3). The comorbidity of depression and cardiovascular disease (CVD) has long been a matter of concern. As a maladaptive psychosocial disorder, depression influences the occurrence and progression of cardiovascular diseases, while CVD can also trigger and worsen depression (4–6). Therefore, there is a need for further exploration of this issue.

Research indicates that inflammation and metabolic factors are common mechanisms underlying both depression and cardiovascular disease. Dysregulation of the innate immune system and adaptive immune system occurs in patients with depression, affecting the treatment and prognosis of depression, including the response to antidepressant medications (7). Furthermore, many metabolic pathways, such as those involving alanine, aspartate, and glutamate metabolism, undergo changes in depression, involving pathways related to inflammation, neuroprotection, and energy metabolism (8). On the other hand, inflammation may serve as a stronger predictor of future cardiovascular events, such as heart attacks and strokes (9). Systemic chronic inflammation (SCI) is the pathological basis of CVD, and it is also an important factor contributing to increased incidence and mortality rates (10).

High-density lipoprotein cholesterol (HDL-C) promotes the efflux of dietary cholesterol through the reverse cholesterol transport pathway and can exert anti-inflammatory and antioxidant effects (11). However, based on current epidemiological evidence, the relationship between HDL-C and depression seems to be difficult to determine. HDL-C levels have been reported to be positively correlated, not correlated, and negatively correlated with depression (12–16). Furthermore, platelets produce a variety of immune effects through the production of numerous cytokines and chemokines after activation (17). Research indicates that patients with depression exhibit platelet overactivation and inflammatory responses (18). However, other studies suggest an association between depression and low platelet activation levels (19). Platelets are closely related to central serotonergic function and are considered a window into brain serotonergic function (20). Disruption of the serotonergic pathway may be associated with platelet aggregation dysregulation in depression and cardiovascular disease (21). These studies all suggest that a single indicator may have unreported deficiencies in predicting depression, and composite indicators may be more promising. The platelet-to-HDL-C ratio (PHR) was initially proposed by Jialal et al. and was considered an effective biomarker for predicting metabolic syndrome (MetS) (22). Since its proposal, research by Ni et al. (23) has indicated a positive non-linear correlation between PHR and the risk of kidney stones. Additionally, a study by Lu et al. (24) supports PHR as an effective marker for non-alcoholic fatty liver disease and liver fibrosis. Based on this, we speculate that, compared to HDL-C, the PHR ratio may be a valuable new predictive signal for depression.

In this study, weighted logistic regression, COX regression, and restricted cubic spline (RCS) regression models were constructed to investigate the association characteristics between PHR and the risk of depression as well as CVD mortality in depressed patients. Receiver operating characteristic (ROC) curves were constructed to evaluate the ability of PHR and HDL-C to predict depression. Additionally, the study explored the mediating role of PHR in a new cardiovascular health (CVH) measure, life’s essential 8 (LE8), in relation to depression.

## 2 Methods

### 2.1 Data source

This cross-sectional study investigated participants in the NHANES survey across 7 cycles from 2005 to 2018. NHANES utilizes a complex, multi-stage probability sampling design, including face-to-face interviews, physical examinations, and laboratory tests for data collection. The survey obtained ethical approval from the National Center for Health Statistics Ethics Review Board, and all participants provided written informed consent. Details are available at https://www.cdc.gov/nchs/nhanes/index.htm. Across the 7 cycles, there were a total of 70,190 participants. Participants were sequentially excluded as follows: 30,441 participants under 20 years old, 3,489 with missing platelet count data, 485 lacking HDL-C testing data, 3,226 with missing depression diagnosis information, and 5,579 participants with missing covariates, resulting in a final eligible sample of 26,970 participants (**Supplementary Figure 1**).

### 2.2 Platelet count/HDL-C (PHR)

The platelet count (PC) (1000 cells/μL) and HDL-C (mmol/L) are biochemical parameters obtained from blood samples collected from participants at the Mobile Examination Center (MEC). The Platelet-to-HDL-C ratio (PHR) was calculated as the ratio of PC to HDL-C (22). Due to the non-normal distribution of PHR values, a standardized transformation (Z-SCORE) was performed.

### 2.3 Depression

Depression was determined based on the Patient Health Questionnaire-9 (PHQ-9), which assesses depressive symptoms present in the past two weeks. The sum of the individual scores for each question (0–3 points) constitutes the depression score, with a total score ranging from 0 to 27 points. A higher score indicates more severe depression. Based on previous research, a PHQ-9 total score of ≥10 is defined as depression (25–27). This definition method of depression is widely used in clinical research, and its sensitivity and specificity are both 88% (25).

### 2.4 Confirming the death status

The National Death Index (NDI) provides death certificate data. NHANES survey data is linked to NDI data using probabilistic matching to ascertain participants’ vital status. Participants for whom NDI matching was unsuccessful are presumed to be alive (28, 29). Follow-up duration is calculated in years based on the time interval between the date of death or December 31, 2019 (whichever came first) and the recruitment date. Cardiovascular disease (CVD) mortality is determined based on the guidelines of the Tenth Revision of the International Statistical Classification of Diseases and Related Health Problems (ICD-10), including 1) Diseases of heart (I00-I09, I11, I13, I20-I51); 2) Cerebrovascular diseases (I60-I69).

### 2.5 Covariates

1) Demographic characteristics: Age (<40 years, 40–60 years, ≥60 years), gender (male/female), race (Mexican American, non-Hispanic Black, non-Hispanic White, other Hispanic, other race - including multiracial), education level (below college, college and above), marital status (divorced/separated/widowed, married/cohabiting, never married) were self-reported at the time of the questionnaire. It also assessed the ratio of household income to poverty level (<1.3, 1.3–3.5, >3.5). 2) Lifestyle habits and physical measures: Alcohol consumption (never, former, current), smoking (never, former, current), total energy intake from the diet (categorized as high or low based on median values), Body Mass Index (BMI), defined as weight in kilograms divided by the square of height in meters (kg/m^2^). 3) Comorbidities: Cancer, liver disease, cardiovascular disease (including coronary artery disease, congestive heart failure, myocardial infarction, stroke, and angina), chronic kidney disease (CKD) (diagnosed as A2, G3a and above for CKD) (30), diabetes, arthritis, thyroid disease. 4) Antidepressant medication use, categorized as non-use and use of antidepressants; antiplatelet medications (including aspirin, dipyridamole, cilostazol, clopidogrel, ticlopidine) and statins (atorvastatin, simvastatin, fluvastatin, lovastatin, nystatin, pravastatin, rosuvastatin, simvastatin).

### 2.6 Life’s Essential 8

LE8 was calculated based on the research conducted by Donald et al. (31). Detailed scoring information for the 8 components can be found in **Supplementary Tables 1** and **2**. In brief, scores were assigned to the 8 sub-item components ranging from 0 to 100, and ultimately LE8 represented the arithmetic mean of these 8 components. The continuous LE8 scores were transformed into a categorical variable with three classes: 0–49 (low), 50–79 (moderate), and 80–100 (high) (32).

### 2.7 Statistical analysis

Following the complex sampling design principles adopted by NHANES, this study utilized Mobile Examination Center (MEC) weights (1/7 * WTMEC2YR) for subsequent analysis. Participants were divided into four groups based on PHR quartiles, with continuous variables analyzed using one-way ANOVA or Kruskal-Wallis tests and results presented as means (standard errors) or medians (IQR); categorical variables were analyzed using chi-square tests and presented as counts (n) and percentages (%). To meet statistical requirements and facilitate interpretation, PHR was standardized (Z-SCORE) or categorized into four groups and included in weighted multivariable logistic regression and COX proportional hazards models to estimate the associations between exposure and outcome risks using odds ratios (ORs), hazard ratios (HRs), and 95% confidence intervals (CIs). Multiple models were constructed: Model 0, unadjusted; Model 1, adjusted for gender, age, race, and education; Model 2, further adjusted for marital status, household income to poverty ratio, alcohol consumption, smoking, and BMI; Model 3, further adjusted for comorbidities, antidepressant medication use, antiplatelet use and statins use. In the survival analysis model, due to the limited number of CVD-specific deaths among depressed survivors, we employed Least Absolute Shrinkage and Selection Operator regression (LASSO) to select important covariates and simplify the model. Collinearity was assessed using variance inflation factor (VIF), with all VIF values being below 10 in this study; Schoenfeld residuals were used to test the proportional hazards assumption. Subgroup analyses were conducted, exploring interactions between covariates and PHR using likelihood ratio tests. Exposure-response relationships between continuous PHR and risk of depression or CVD mortality risk among depressed patients were evaluated using restricted cubic splines (RCS) with 3 nodes (10th, 50th, and 90th percentile). Additionally, in the mediation analysis, the Bootstrap method was employed to estimate the mediation effect and its confidence intervals through 5000 repeated simulations (27, 33–35).

Several sensitivity analyses were conducted: 1) without considering weights; 2) multiple imputation of missing covariates; 3) additional adjustment for metabolic syndrome; 4) in the survival analysis, individuals who died within two years of follow-up were excluded.

All statistical procedures involved in this study were performed using R software version 4.3.1 (R Foundation for Statistical Computing). The “survey” package and “survival” package were used for weighted regression analysis; the “rms” package was utilized to build RCS regression models; the “mediation” package was employed for mediation analysis; the “mice” package was used for multiple imputation; the “glmnet” package was used to implement LASSO regression for selecting feature variables in survival analysis; and the “car” package was used to calculate variance inflation factors. In the present study, a two-tailed p-value < 0.05 was considered statistically significant.

## 3 Results

### 3.1 Population characteristics

A total of 26,970 eligible participants were included after strict exclusion criteria (**Supplementary Figure 1**), among whom 2,308 were diagnosed with depression, resulting in a weighted prevalence of 7.45%. **Table 1** presents detailed data on PHR, HDL-C, and PC for all participants. **Supplementary Table 3** presents the characteristics of each group based on quartiles. Compared to the lowest quartile group of PHR, individuals in the highest quartile group had an approximately 65% higher prevalence of depression, with more males, younger age, higher proportion of Mexican ethnicity, lower education level, higher unmarried rate, higher poverty level, higher past alcohol consumption and current smoking, higher overweight or obesity rate, and higher energy intake. In terms of comorbidities, the prevalence of cancer, thyroid disease, liver disease, arthritis, CVD, and CKD was lower, while the prevalence of diabetes was higher. Additionally, the individuals in the highest quartile group had a lower proportion of using statins. In the survival analysis, during a mean follow-up period of 7.57 years, until December 31, 2019, 289 deaths were identified among the 2,308 depressed patients, with 83 of them being CVD-specific deaths. Among individuals who died due to CVD-specific causes, there was a higher age and, higher proportion of low education level, and the Divorced/separated/widowed category was twice as high as the survivor group. The proportion of current alcohol drinkers was lower. Furthermore, there was a 2–3 times higher probability of comorbid arthritis, diabetes, CVD, CKD, and a higher proportion of statins and antiplatelet use (**Supplementary Table 4**).

**Table 1:** The unweighted statistical values of PHR, HDL-C, and PC.

				Percentiles					
	Mean	SD	Min	P 1	P 25	P 50	P 75	P 99	Max
PC (1000 cells/μL)	248.3	66.386	11	118	204	241	285	443.31	1000
HDL-C (mmol/L)	1.367	0.415	0.16	0.67	1.06	1.29	1.6	2.61	5.84
PHR	184.328	81.813	9.244	69.604	141.606	184.328	238.542	449.559	1835.185

HDL-C, high-density lipoprotein cholesterol; PHR, platelet-to-high-density lipoprotein cholesterol ratio; PC, platelet count; SD, standard deviation.

### 3.2 Estimation of the association of PHR with depression and CVD mortality among depression patients

**Figure 1:**
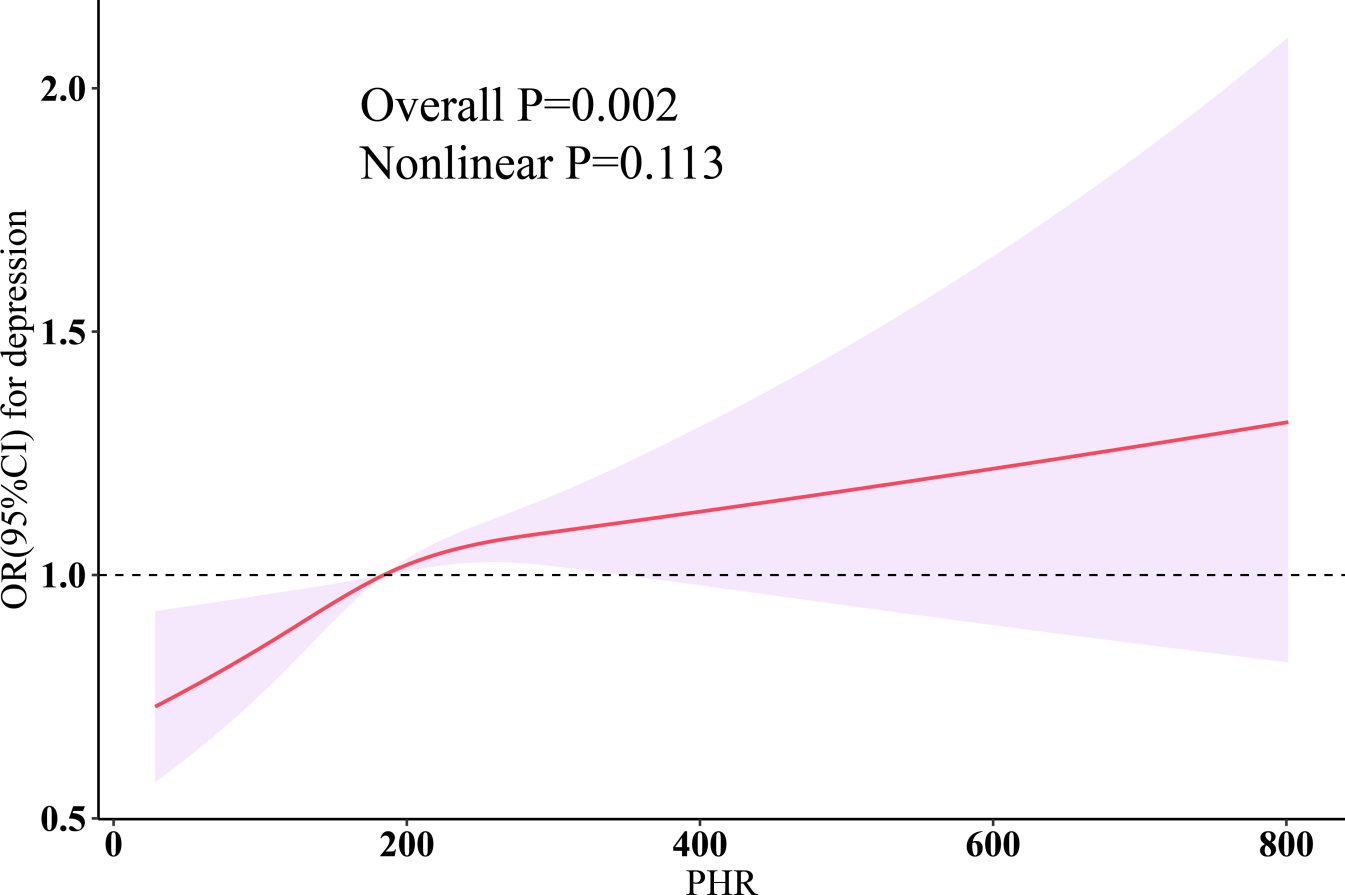
Dose-response relationship of PHR with depression. Three knots (10th, 50th, 90th percentiles) were selected for fitting the restricted cubic spline model, and the median value of PHR (184.328) was served as the reference point. All models were adjusted for age, sex, ethnicity, education, marital status, poverty-income ratio, total energy intake, BMI, smoking, drinking, arthritis, thyroid problems, cancer, diabetes, liver diseases, CVD, CKD, statins, and antiplatelet. HDL-C, high-density lipoprotein cholesterol; PHR, platelet-to-high-density lipoprotein cholesterol ratio; BMI, body mass index; CVD, cardiovascular disease; CKD, chronic kidney disease; OR, odds ratio; CI, confidence interval.

The results of weighted multivariable logistic regression are shown in **Table 2**. In the crude model (Model 0), a per SD increase in PHR [OR (95% CI): 1.20 (1.14, 1.25), P < 0.0001] was associated with an increased risk of depression. After fully adjusting for depression risk factors, we estimated that the odds ratios (ORs) of depression associated with a per SD increase in PHR were 1.06 (95% CI: 1.01–1.12, P = 0.03). When PHR was included in the model as a categorical variable and after full adjustment for confounders, compared to the lowest quartile group, participants in quartile 2 [OR (95% CI): 1.20 (0.99, 1.46), P = 0.07], quartile 3 [OR (95% CI): 1.24 (1.03, 1.50), P = 0.03], and quartile 4 [OR (95% CI): 1.31 (1.10, 1.56), P = 0.003] showed a trend of increasing risk of depression (p trend = 0.01). Restricted cubic splines (RCS) demonstrated a linear relationship between PHR and depression (Nonlinear P = 0.113) (**Figure 1**).

**Table 2:** Weighted multivariate Logistic regression analysis for the risk of depression with PHR.

PHR		Model 0		Model 1		Model 2		Model 3	
Categorical		OR (95% CI)	P	OR (95% CI)	P	OR (95% CI)	P	OR (95% CI)	P
	Quartile 1	ref		ref		ref		ref	
	Quartile 2	1.25(1.04,1.50)	0.02	1.27(1.06,1.52)	0.01	1.17(0.98,1.41)	0.09	1.20(0.99,1.46)	0.07
	Quartile 3	1.43(1.19,1.70)	<0.001	1.42(1.19,1.71)	<0.001	1.24(1.03,1.48)	0.02	1.24(1.03,1.50)	0.03
	Quartile 4	1.71(1.46,2.02)	<0.0001	1.67(1.41,1.98)	<0.0001	1.34(1.12,1.60)	0.002	1.31(1.10,1.56)	0.003
	p for trend		<0.0001		<0.0001		0.003		0.01
Continuous									
	per SD^+^	1.20(1.14,1.25)	<0.0001	1.18(1.13,1.24)	<0.0001	1.09(1.03,1.15)	0.002	1.06(1.01,1.12)	0.03

Model 0: Not adjusted;

Model 1: Adjusted for age, gender, education attainment, and race;

Model 2: Further adjusted for marital status, poverty-income ratio, smoking and drinking status, BMI and total energy intake based on Model 1;

Model 3: Further adjusted for arthritis, thyroid problems, cancer, diabetes, liver diseases, CVD, CKD, statins, and antiplatelet based on Model 2.

PHR, platelet-to-high-density lipoprotein cholesterol ratio; BMI, body mass index; CVD, cardiovascular disease; CKD, chronic kidney disease; SD, standard deviation; OR, odds ratio; CI, confidence interval; ref, reference.

**Figure 2:**
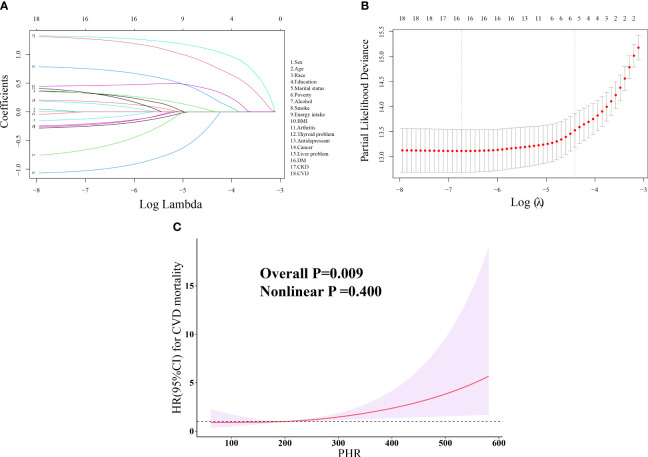
LASSO regression of the 18 covariates associated with CVD mortality and Dose-response relationship between PHR and CVD mortality among depression patients. **(A)** The screening path corresponds to 18 covariates that contribute to CVD mortality among depression patients. **(B)** The association between the log-transformed λ and Partial Likelihood Deviance for CVD mortality among depression patients. The red dashed line and its error bars represent the average Partial Likelihood Deviance value and the corresponding 95% CI. The left black dashed line represents the optimal λ value obtained when calculating the minimum Partial Likelihood Deviance, while the right black dashed line represents the λ value of the simplest model obtained at one standard error from the minimum Partial Likelihood Deviance. **(C)** Dose-response relationship between PHR and CVD mortality among depression patients. Three knots (10th, 50th, 90th percentiles) were selected for fitting the restricted cubic spline model, and the median value of PHR (198.54) was served as the reference point, and models were adjusted for age, race, BMI, DM, CVD CKD, statins, and antiplatelet. HDL-C, high-density lipoprotein cholesterol; PHR, platelet-to-high-density lipoprotein cholesterol ratio; BMI, body mass index; DM, diabetes; CVD, cardiovascular disease; CKD, chronic kidney disease; LASSO, least absolute shrinkage and selection operator; HR, hazard ratio; CI, confidence interval.

Considering that there were only 83 cases of CVD-specific deaths, LASSO regression was used for variable selection and identified a set of important variables, including Age, race, BMI, DM, CKD, and CVD, totaling 6 factors (**Figures 2A, B**). A multivariable COX regression model was then constructed based on these variables. Schoenfeld residual tests in this study indicated that all models met the proportional hazards assumption. As shown in **Table 3**, weighted COX regression demonstrated that for PHR, in the unadjusted model (Model 0), per SD increase in PHR was not significantly associated with CVD mortality [HR (95% CI): 1.15 (0.85, 1.54), P = 0.36]; after adjusting for the above 6 factors, statins, and antiplatelet, we estimated that a per SD increase in PHR was associated with a higher risk of CVD mortality among depression survivors (HR: 1.38, 95% CI: 1.05–1.81, P = 0.02), and RCS fitted a linear dose-response relationship between PHR and CVD mortality (Nonlinear P = 0.400) (**Figure 2C**).

**Table 3:** Weighted multivariate Cox regression analysis for the risk of PHR with CVD mortality among depression patients.

	Model 0		Model 1	
	HR (95% CI)	P	HR (95% CI)	P
PHR (per SD^+^)	1.15(0.85,1.54)	0.36	1.38(1.05, 1.81)	0.02
PHR (per 100^+^)	1.17(0.84,1.63)	0.36	1.44(1.06, 1.95)	0.02

Model 0: Not adjusted;

Model 1: Adjusted for age, race, BMI, DM, CVD, CKD, statins, and antiplatelet.

PHR, platelet-to-high-density lipoprotein cholesterol ratio; CVD, cardiovascular disease; BMI, body mass index; DM, diabetes; CKD, chronic kidney disease; SD, standard deviation; HR, hazard ratio; CI, confidence interval.

**Figure 3:**
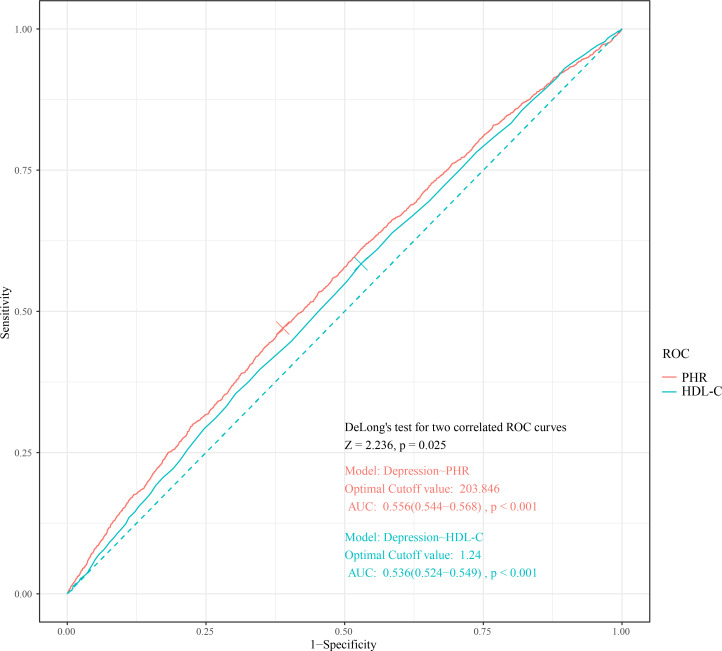
Unadjusted ROC curves to predict depression for PHR and HDL-C. ROC, receiver operating characteristic; HDL-C, high-density lipoprotein cholesterol; PHR, platelet-to-high-density lipoprotein cholesterol ratio.

The areas under the ROC curves (AUC) were 0.556 (95% CI, 0.544–0.568; P < 0.001) for PHR and 0.536 (95% CI, 0.524–0.549; P < 0.001) for HDL-C (PDeLong = 0.025) (**Figure 3**).

### 3.3 Subgroup analysis and sensitivity analysis

The current alcohol consumption (P for interaction: 0.014) was observed to enhance the association between PHR and depression (**Supplementary Figure 2**). Additionally, the associations between PHR and CVD mortality in depression survivors were generally consistent across all subgroups (**Supplementary Figure 3**). Furthermore, the results of several sensitivity analyses were consistent with the main results (**Supplementary Tables 5**, **6**).

### 3.4 Mediation analysis

**Figure 4:**
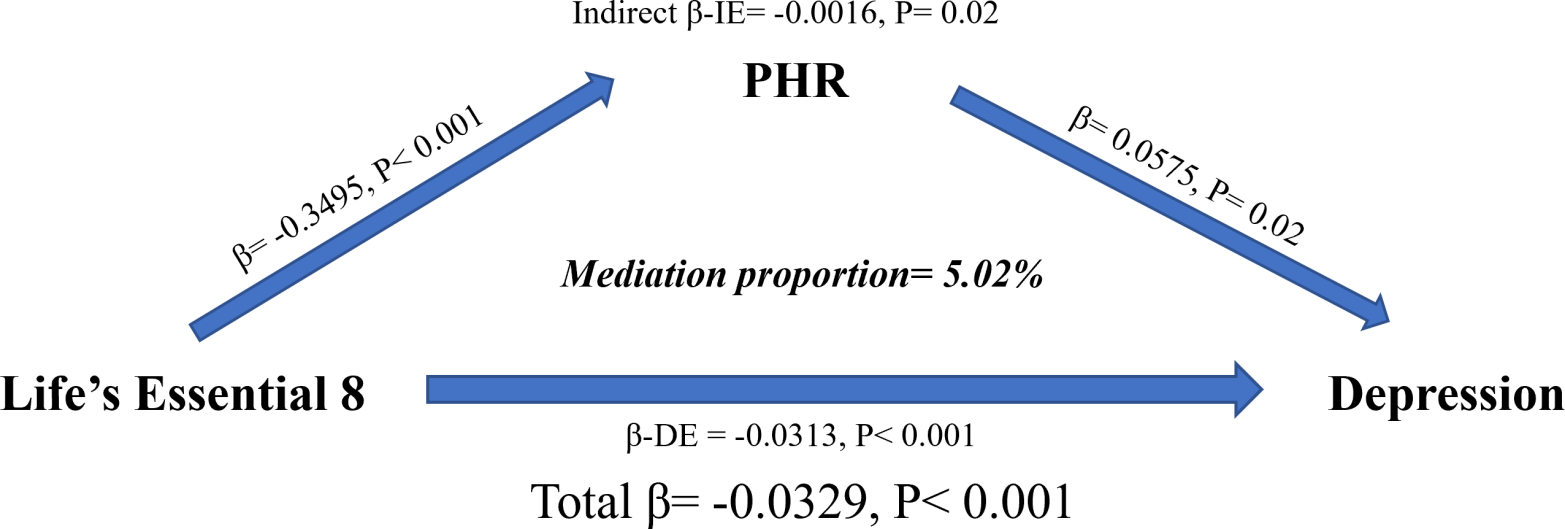
Estimated proportion of the association between LE8 and depression mediated by PHR. All models were adjusted for age, sex, ethnicity, education, marital status, poverty-income ratio, total energy intake, BMI, smoking, drinking, arthritis, thyroid problems, cancer, diabetes, liver diseases, CVD, CKD, statins, and antiplatelet. Mediation proportion = β-IE/Total β. IE, indirect effect; DE, direct effect; PHR, platelet-to-high-density lipoprotein cholesterol ratio; LE8, life’s essential 8; BMI, body mass index; CVD, cardiovascular disease; CKD, chronic kidney disease.

As is shown in **Figure 4**, we estimated per grade increase in LE8 was significantly negatively correlated with PHR (β= -0.3495, P < 0.001); per SD increase in PHR was associated with depression (β= -0.0575, P = 0.02); and mediation analysis revealed that PHR mediated 5.02% of the association between LE8 and depression.

## 4 Discussion

In this large-scale nationally representative observational study, our findings provide strong evidence supporting a linear increase in depression risk with PHR. Current alcohol consumption significantly enhances the association between PHR and depression. Furthermore, compared to HDL-C, PHR has a better predictive ability for depression. Additionally, there is a positive linear dose-response relationship between PHR and CVD mortality in patients with depression. Sensitivity analyses, including without considering weights, multiple imputations of missing covariates, additional adjustment for metabolic syndrome, and excluding individuals who died within two years of follow-up in survival analysis, all support our findings. Finally, mediation analysis shows that maintaining good cardiovascular health significantly prevents the occurrence of depression by reducing PHR. In conclusion, our results suggest that PHR is an intermediate factor between cardiovascular disease and depression. On one hand, higher PHR in patients with depression is associated with a higher risk of cardiovascular death. On the other hand, maintaining good cardiovascular health can reduce the risk of depression through PHR reduction.

Previously, a large-scale retrospective study from Beijing, China found no difference in Platelet-to-HDL ratio (PHR) between Unipolar depression (UD), bipolar depression (BD), and healthy controls (HC). However, in a multivariate logistic regression model adjusting for confounding factors, a positive correlation between PHR and Unipolar depression (UD) was observed (36). In our study, although the association between PHR and depression gradually decreased after considering confounding factors, PHR remained significantly associated with depression in all models. PHR is defined as the ratio of platelet count (PC) to HDL-C concentration (22, 37). Platelets play a crucial role in inflammation, as they produce numerous cytokines and chemokines when activated, leading to various immune effects (17). Platelets are considered a potential intermediate factor linking mental illness and inflammatory responses (38–40). Studies indicate that patients with depression exhibit platelet over-activation and inflammatory responses (18). However, there are also findings suggesting a low level of platelet activation in depression (19). Thus, further evidence is needed to clarify the role of platelets in depression. The relationship between HDL-C and depression is complex. Lower serum HDL-C levels are associated with a higher risk of depression or more severe depressive symptoms (41–45). Conversely, some studies suggest that higher HDL-C levels are correlated with more severe depressive symptoms (46, 47). These contradictory findings imply a more intricate dynamic regulatory mechanism, emphasizing the value of this study. Furthermore, cardiovascular disease patients are more prone to depression compared to the general population. Individuals with depression are more likely to develop cardiovascular disease, with higher mortality rates, possibly linked to increased inflammatory markers (48–51). Our results support PHR as a novel inflammation marker that significantly predicts CVD mortality in patients with depression. Additionally, through mediation analysis, we established that better cardiovascular health prevents depression by reducing PHR, further supporting the interplay of PHR in depression and CVD. However, the specific mechanistic relationship of PHR requires further elucidation.

Subgroup analysis showed a significant interaction between current alcohol consumption and PHR. Alcohol is a central nervous system depressant that slows down the activity of the central nervous system. Long-term alcohol consumption can lead to structural abnormalities in the brain and increase the risk of depression (52, 53). In a large multi-ethnic cohort, increasing levels of alcohol consumption were associated with higher levels of all HDL markers. Mild drinkers had lower levels of HDL markers compared to moderate drinkers (54). Huang et al. (55) also found that moderate alcohol consumption may be associated with a slower decrease in HDL-C, but excessive alcohol consumption may have a negative impact on HDL-C. On the other hand, a relationship has been established between alcohol consumption frequency and quantity and platelet count reduction (56). Wine, particularly its phenolic compounds, seems to have a more pronounced inhibitory effect on platelet aggregation (57).

## 5 Advantages and limitations

To our knowledge, this is the first observational study to explore the relationship between PHR and depression in the entire adult population of the United States; representing a weighted sample of 160 million adult individuals. Furthermore, our study comprehensively considered risk factors for depression, therefore, the conclusions are relatively reliable. Lastly, considering that HDL-C and platelet count are routine indicators that are easily accessible, they may have significant potential in the diagnosis of depression and cardiovascular prognosis assessment in depressed populations. However, there are some limitations to consider. First, regarding the relationship between PHR, HDL-C, and depression, further validation is needed in prospective studies involving larger cohorts due to the cross-sectional design. Second, HDL-C comprises multiple subgroups with different biological characteristics, but in this study, we did not have data on the quantity and size of HDL subgroups or particles. Third, potential confounding factors were inevitably overlooked. Fourth, depression diagnosis was based on self-reporting, which could be prone to bias. Fifth, depression is related to platelet function, yet this study did not obtain data on platelet functional parameters (58, 59). Sixth, this study assessed HDL-C concentration and platelet count based on a single measurement, which could introduce potential bias. Seventh, some treatments may affect the association between PHR and depression; however, in the current study, more detailed exploration cannot be carried out. Finally, it must be admitted that although the use of antidepressant drugs has been adjusted, some patients may have received effective treatment and therefore will not be diagnosed with depression during the investigation. To reduce this potential bias, we redefine depression. In additional analysis 1, using antidepressants or PHQ-9 ≥ 10 was defined as depression to define some potential depressive populations as depression. In additional analysis 2: Not using antidepressants and having PHQ-9 ≥ 10 was defined as depression to explore the association between PHR and untreated depression. In additional analysis 3, after excluding the participants with PHQ-9 ≥ 10 score, the individuals who use antidepressants and have PHQ-9 < 10 was defined as depression to explore the association between PHQ-9 and the effectively treated depression. In additional analysis 4: we exclude the people who use antidepressants to rule out the interference of antidepressants. Reassuringly, all four additional analyses above further confirmed the robust association of PHR with depression (**Supplementary Table 7**).

## 6 Conclusion

In conclusion, our study identified the bridging role of PHR in depression and CVD among U.S. adults. PHR appears to be a potential mechanism for the co-occurrence of these two conditions, and further validation of our findings is needed in more clinical trials and mechanistic studies in the future.

## Data availability statement

Publicly available datasets were analyzed in this study. This data can be found here: https://www.cdc.gov/nchs/nhanes/index.htm.

## Ethics statement

The studies involving humans were approved by The National Center for Health Statistics institutional review board. The studies were conducted in accordance with the local legislation and institutional requirements. The participants provided their written informed consent to participate in this study.

## Author contributions

HZ: Conceptualization, Formal analysis, Methodology, Software, Writing – original draft, Writing – review & editing. YX: Formal analysis, Investigation, Methodology, Software, Validation, Visualization, Writing – review & editing. YYX: Conceptualization, Investigation, Methodology, Supervision, Validation, Writing – review & editing.
