# Supplementary material for: The value of the platelet/high-density lipoprotein cholesterol ratio in predicting depression and its cardiovascular disease mortality: a population-based observational study
Source: Front Endocrinol (Lausanne). 2024 Jul 29;15:1402336. doi: 10.3389/fendo.2024.1402336 (PMC11325088; doi:10.3389/fendo.2024.1402336)
Supplement: Supplementary file 1 [file DataSheet_1.docx]

# Supplementary Figures

## Supplementary Figure 1: Flow chart for inclusion of participants


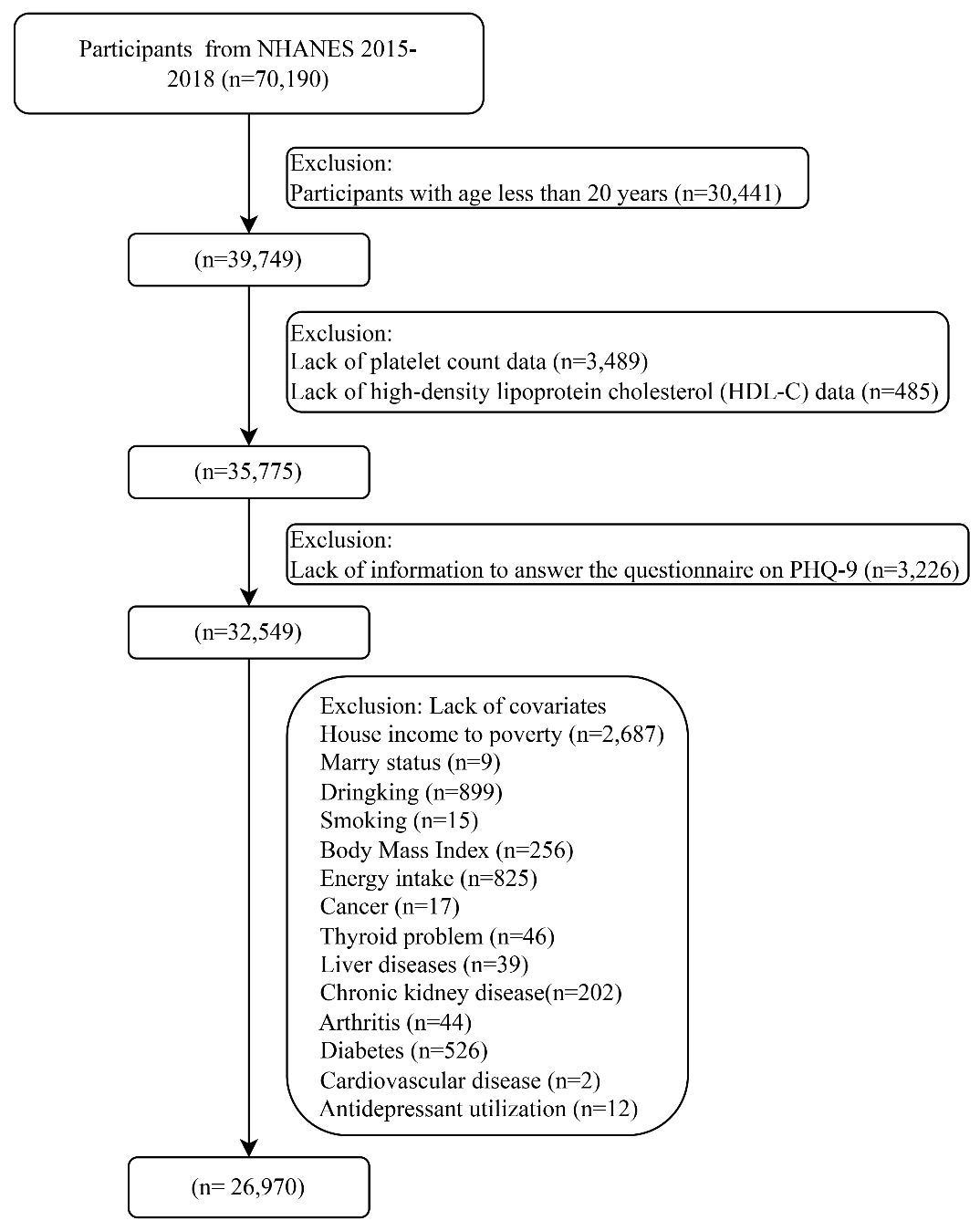


## Supplementary Figure 2: Forest plot of subgroup analysis and interaction tests for the association between PHR and depression

**Figure legend:** All models adjusted for 19 risk factors other than stratification variables, and the significance of the interaction was determined by the likelihood ratio test.

**Abbreviation:** PHR, platelet-to-high-density lipoprotein cholesterol ratio; CVD, cardiovascular disease; CKD, chronic kidney disease; OR, odds ratio; CI, confidence interval; P int, p for interaction.


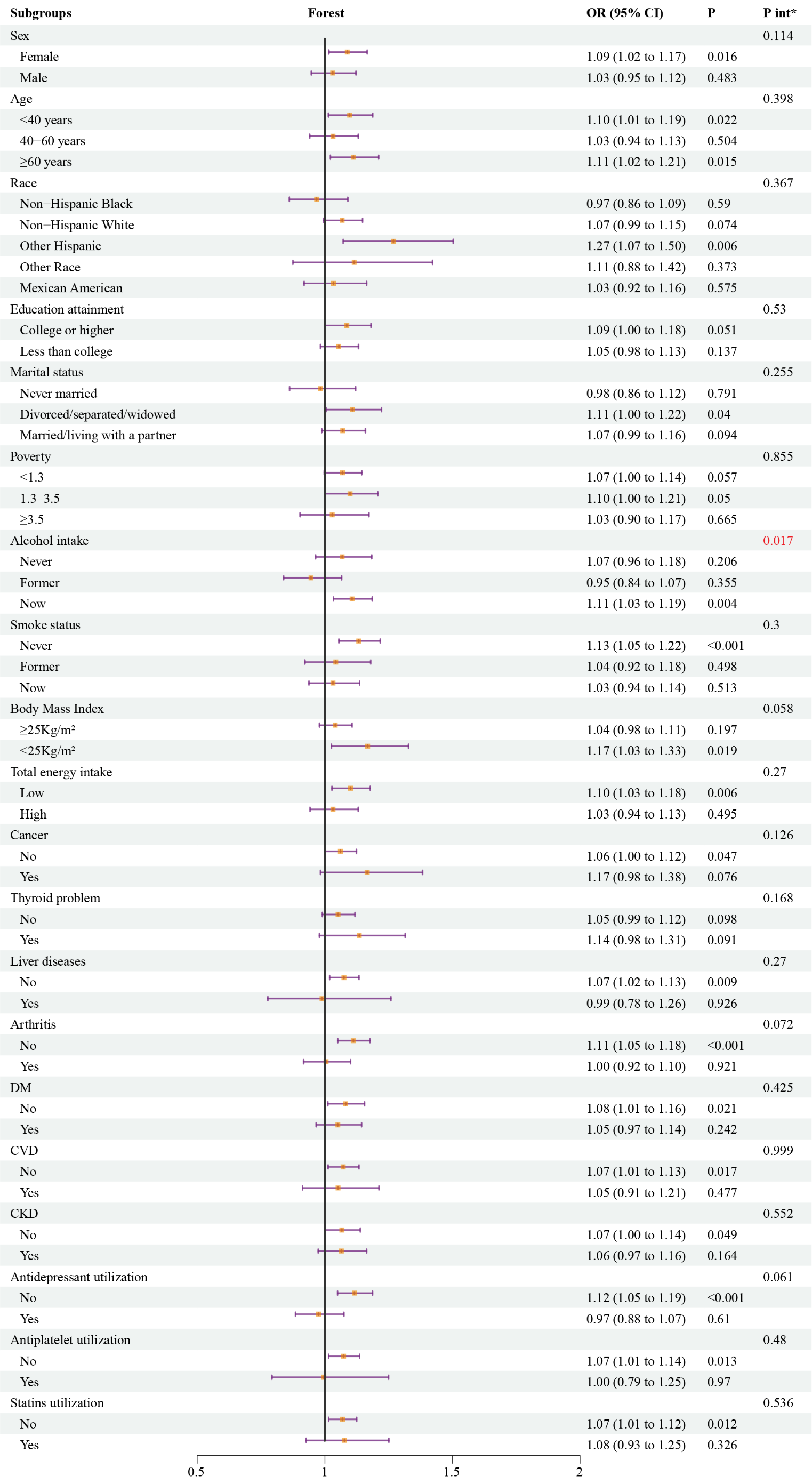


## Supplementary Figure 3: Forest plot of subgroup analysis and interaction tests for the association between PHR and CVD mortality among depression patients

**Figure legend:** All models adjusted for 7 risk factors other than stratification variables, and the significance of the interaction was determined by the likelihood ratio test.

**Abbreviation:** PHR, platelet-to-high-density lipoprotein cholesterol ratio; DM, diabetes; CVD, cardiovascular disease; CKD, chronic kidney disease; HR, hazard ratio; CI, confidence interval; P int, p for interaction.


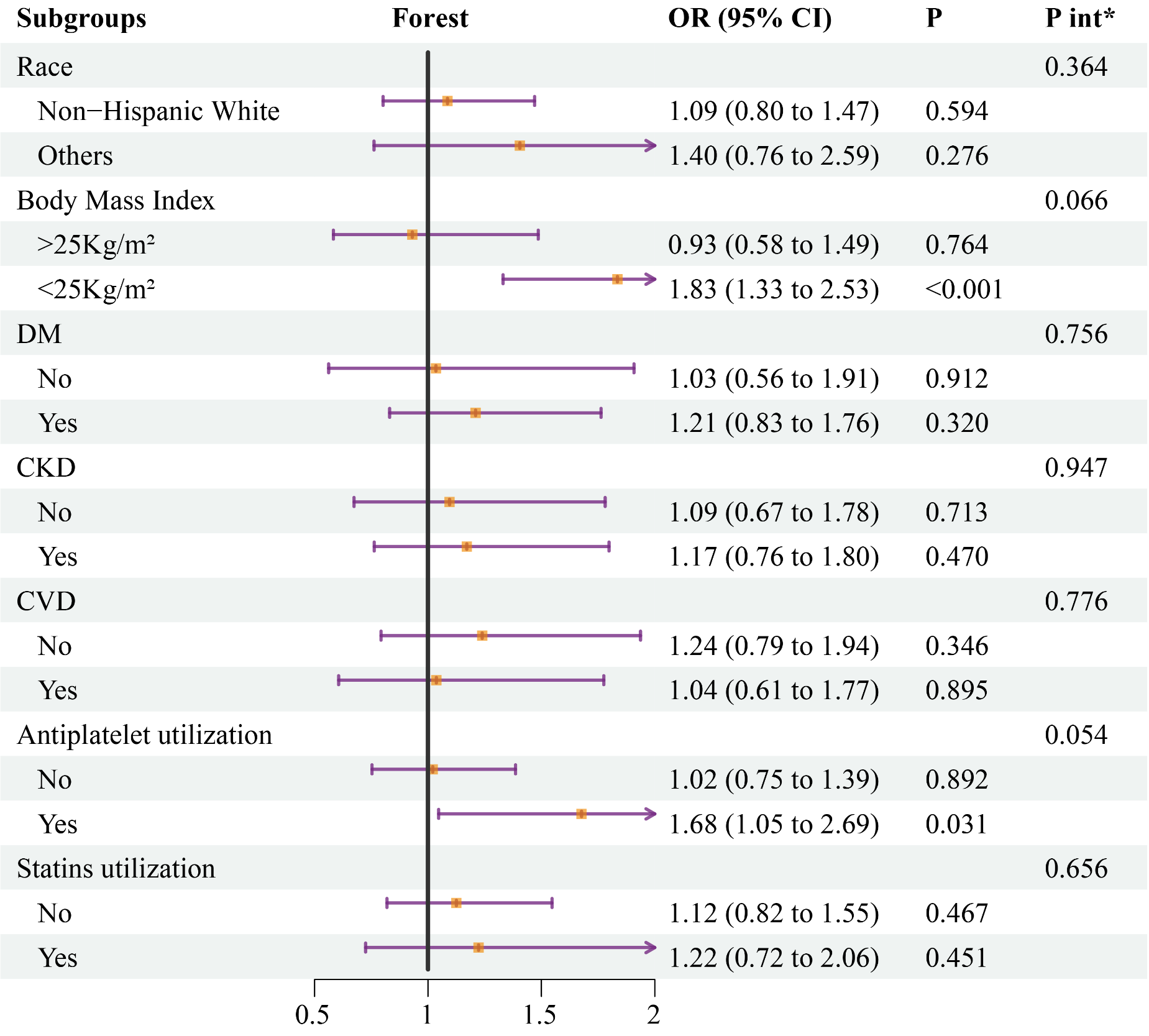


# Supplementary Tables

## Supplementary Table 1: Detailed information of Healthy Eating Index -2015 (HEI-2015)

for good health).

^2^Includes 100% fruit juice.

^3^Includes all forms except juice.

^4^Includes legumes (beans and peas).

^5^Includes all milk products, such as fluid milk, yogurt, and cheese, and fortified soy beverages.

^6^Includes legumes (beans and peas).

^7^Includes seafood, nuts, seeds, soy products (other than beverages), and legumes (beans and peas).

^8^Ratio of poly- and monounsaturated fatty acids (PUFAs and MUFAs) to saturated fatty acids (SFAs).

**Reference:**  Krebs-Smith, S.M., Pannucci, T.E., Subar, A.F., Kirkpatrick, S.I., Lerman, J.L., Tooze, J.A., Wilson, M.M., Reedy, J., 2018. Update of the Healthy Eating Index: HEI-2015. Journal of the Academy of Nutrition and Dietetics 118, 1591–1602. <https://doi.org/10.1016/j.jand.2018.05.021>

| Component | Maximum points | Standard for maximum score | Standard for minimum score of zero |
| --- | --- | --- | --- |
| Adequacy |  |  |  |
| Total fruits^2^ | 5 | ≥ 0.8 cup equivalents per 1,000 kcal | No fruits |
| Whole fruits^3^ | 5 | ≥ 0.4 cup equivalents per 1,000 kcal | No whole fruits |
| Total vegetables^4^ | 5 | ≥ 1.1 cup equivalents per 1,000 kcal | No vegetables |
| Greens and beans^4^ | 5 | ≥ 0.2 cup equivalents per 1,000 kcal | No dark green vegetables or legumes |
| Whole grains | 10 | ≥ 1.5 oz equivalents per 1,000 kcal | No whole grains |
| Dairy^5^ | 10 | ≥ 1.3 cup equivalents per 1,000 kcal | No dairy |
| Total protein foods^6^ | 5 | ≥ 2.5 oz equivalents per 1,000 kcal | No protein foods |
| Seafood and plant proteins^6,7^ | 5 | ≥ 0.8 oz equivalents per 1,000 kcal | No seafood or plant proteins |
| Fatty acids^8^ | 10 | (PUFAs + MUFAs)/SFAs ≥ 2.5 | (PUFAs + MUFAs)/SFAs ≤ 1.2 |
| Moderation |  |  |  |
| Refined grains | 10 | ≤ 1.8 oz equivalents per 1,000 kcal | ≥ 4.3 oz equivalents per 1,000 kcal |
| Sodium | 10 | ≤ 1.1 gram per 1,000 kcal | ≥ 2.0 grams per 1,000 kcal |
| Added sugars | 10 | ≤ 6.5% of energy | ≥ 26% of energy |
| Saturated fats | 10 | ≤ 8% of energy | ≥ 16% of energy |

## Supplementary Table 2: Criteria for scoring of Life’s Essential 8

| **Domain** | **CVH metric** | **Method of measurement** | **Quantification of CVH metric** | **Score, points** |
| --- | --- | --- | --- | --- |
| Health Behaviors | Diet | Quantiles of HEI-2015 (population)  Example tools: 24-hour dietary recall | 1st–24th | 0 |
|  |  |  | 25th–49th | 25 |
|  |  |  | 50th–74th | 50 |
|  |  |  | 75th–94th | 80 |
|  |  |  | ≥95th | 100 |
|  | PA | Self-reported minutes of moderate or vigorous PA per week  Example tools: NHANES PAQ | 0 | 0 |
|  |  |  | 1–29 | 20 |
|  |  |  | 30–59 | 40 |
|  |  |  | 60–89 | 60 |
|  |  |  | 90–119 | 80 |
|  |  |  | 120–149 | 90 |
|  |  |  | ≥150 | 100 |
|  | Nicotine  exposure | Self-reported use of cigarettes or inhaled  NDS  Example tools: NHANES SMQ and SMQFAM | Current smoker | 0 |
|  |  |  | Former smoker quit <1 y, or currently using inhaled NDS, with active indoor smoker | 5 |
|  |  |  | Former smoker, quit <1 y, or currently using inhaled NDS, without active indoor smoker | 25 |
|  |  |  | Former smoker, quit 1–<5 y, with active indoor smoker | 30 |
|  |  |  | Former smoker, quit 1–<5 y, without active indoor smoker | 50 |
|  |  |  | Former smoker, quit ≥5 y, with active indoor smoker | 55 |
|  |  |  | Former smoker, quit ≥5 y, without active indoor smoker | 75 |
|  |  |  | Never smoker, with active indoor smoker | 80 |
|  |  |  | Never smoker, without active indoor smoker | 100 |
|  | Sleep health | Self-reported average hours of sleep per night  Example tools: NHANES SLQ | <4 | 0 |
|  |  |  | 4–<5 | 20 |
|  |  |  | 5–<6 or ≥10 | 40 |
|  |  |  | 6–<7 | 70 |
|  |  |  | 9–<10 | 90 |
|  |  |  | 7–<9 | 100 |
| Health factors | BMI | Body weight (kilograms) divided by height squared (meters squared)   1. Example tools: NHANES BMX | ≥40.0 | 0 |
|  |  |  | 35.0–39.9 | 15 |
|  |  |  | 30.0–34.9 | 30 |
|  |  |  | 25.0–29.9 | 70 |
|  |  |  | <25 | 100 |
|  | Blood lipids | Plasma total and HDL cholesterol with calculation of non–HDL cholesterol   1. Example tools: NHANES TCHOL, HDL and BPQ | ≥220 or 190–219 (take medication) | 0 |
|  |  |  | 190–219(no medication) or 160–189 (take medication) | 20 |
|  |  |  | 160–189(no medication) or 130–159(take medication) | 40 |
|  |  |  | 130–159(no medication) | 60 |
|  |  |  | <130(take medication) | 80 |
|  |  |  | <130(no medication) | 100 |
|  | Blood glucose | Casual HbA1c (%) | Diabetes with HbA1c ≥10.0 | 0 |
|  |  |  | Diabetes with Hb A1c 9.0–9.9 | 10 |
|  |  |  | Diabetes with HbA1c 8.0–8.9 | 20 |
|  | Blood glucose | Casual HbA1c (%)  Example tools: NHANES GHB and DIQ | Diabetes with HbA1c 7.0–7.9 | 30 |
|  |  |  | Diabetes with HbA1c <7.0 | 40 |
|  |  |  | No diabetes and HbA1c 5.7–6.4) | 60 |
|  |  |  | No history of diabetes and HbA1c <5.7 | 100 |
|  | BP | Appropriately measured systolic and diastolic BPs  Example tools: NHANES BPX and BPQ | ≥160 or ≥100 | 0 |
|  |  |  | 140–159 or 90–99(take medication) | 5 |
|  |  |  | 140–159 or 90–99(no medication) | 25 |
|  |  |  | 130–139 or 80–89(take medication) | 30 |
|  |  |  | 130–139 or 80–89(no medication) | 50 |
|  |  |  | 120–129/<80 (take medication) | 55 |
|  |  |  | 120–129/<80 (no medication) | 75 |
|  |  |  | <120/<80(take medication) | 80 |
|  |  |  | <120/<80(no medication) | 100 |

## Supplementary Table 3: Weighted characteristics of the eligible 26,970 participants in the analysis of PHR with the risk of depression

| Characteristics | Total [9.24,1835.19] | Quartile 1 [9.24,141.61] | Quartile 2 (141.61,184.33] | Quartile 3 (184.33,238.54] | Quartile 4 (238.54,1835.19] | P value |
| --- | --- | --- | --- | --- | --- | --- |
| Platelet (1000 cells/μL), Mean (S.E) | 248.90(0.80) | 200.59(0.89) | 233.52(0.73) | 258.02(0.84) | 305.62(1.24) | < 0.0001 |
| HDL-C (mmol/L), Mean (S.E) | 1.38(0.01) | 1.82(0.01) | 1.44(0.00) | 1.24(0.00) | 1.02(0.00) | < 0.0001 |
| Depression, n (%) |  |  |  |  |  | < 0.0001 |
| No | 24662(92.55) | 6295(94.33) | 6196(93.02) | 6152(92.11) | 6019(90.66) |  |
| Yes | 2308(7.45) | 449(5.67) | 541(6.98) | 597(7.89) | 721(9.34) |  |
| Sex, n (%) |  |  |  |  |  | < 0.0001 |
| Female | 13450(50.57) | 3781(59.93) | 3377(50.55) | 3162(46.31) | 3130(45.29) |  |
| Male | 13520(49.43) | 2963(40.07) | 3360(49.45) | 3587(53.69) | 3610(54.71) |  |
| Age, n (%) |  |  |  |  |  | < 0.0001 |
| <40 years | 9042(36.05) | 1764(28.22) | 2199(35.56) | 2336(38.04) | 2743(42.61) |  |
| 40−60 years | 9090(38.69) | 2057(36.49) | 2150(37.23) | 2414(40.52) | 2469(40.61) |  |
| ≥60 years | 8838(25.27) | 2923(35.29) | 2388(27.21) | 1999(21.45) | 1528(16.78) |  |
| Race, n (%) |  |  |  |  |  | < 0.0001 |
| Mexican American | 4129(7.94) | 730(5.28) | 934(7.04) | 1154(8.82) | 1311(10.73) |  |
| Non-Hispanic Black | 5494(10.10) | 1688(11.54) | 1372(9.93) | 1276(9.51) | 1158(9.41) |  |
| Non-Hispanic White | 12265(70.28) | 3165(73.73) | 3119(71.72) | 3013(68.98) | 2968(66.53) |  |
| Other Hispanic | 2442(5.00) | 488(3.60) | 599(4.60) | 671(5.62) | 684(6.21) |  |
| Other Races | 2640(6.68) | 673(5.85) | 713(6.70) | 635(7.06) | 619(7.12) |  |
| Education attainment, n (%) |  |  |  |  |  | < 0.0001 |
| Less than college | 12346(37.62) | 2727(31.52) | 2962(35.60) | 3219(39.90) | 3438(43.68) |  |
| College or higher | 14624(62.38) | 4017(68.48) | 3775(64.40) | 3530(60.10) | 3302(56.32) |  |
| Marital status, n (%) |  |  |  |  |  | 0.01 |
| Never married | 4789(17.46) | 1163(15.73) | 1192(17.81) | 1193(17.97) | 1241(18.37) |  |
| Divorced/separated/widowed | 5919(18.20) | 1701(19.91) | 1472(18.22) | 1406(17.86) | 1340(16.75) |  |
| Married/living with a partner | 16262(64.34) | 3880(64.36) | 4073(63.96) | 4150(64.17) | 4159(64.88) |  |
| Poverty, n (%) |  |  |  |  |  | < 0.0001 |
| <1.3 | 8138(19.95) | 1742(15.94) | 1916(19.03) | 2112(20.55) | 2368(24.46) |  |
| 1.3–3.5 | 10207(35.51) | 2570(34.60) | 2506(34.05) | 2570(36.56) | 2561(36.89) |  |
| >3.5 | 8625(44.54) | 2432(49.47) | 2315(46.91) | 2067(42.88) | 1811(38.65) |  |
| Alcohol status, n (%) |  |  |  |  |  | < 0.0001 |
| Never | 3580(10.20) | 897(9.84) | 869(9.62) | 899(10.59) | 915(10.80) |  |
| Former | 4379(13.15) | 942(10.19) | 997(12.02) | 1146(13.92) | 1294(16.61) |  |
| Now | 19011(76.65) | 4905(79.97) | 4871(78.36) | 4704(75.49) | 4531(72.60) |  |
| Smoke, n (%) |  |  |  |  |  | < 0.0001 |
| Never | 14694(54.88) | 3841(57.25) | 3722(55.88) | 3659(54.34) | 3472(51.95) |  |
| Former | 6669(25.07) | 1772(27.35) | 1749(26.29) | 1628(24.05) | 1520(22.46) |  |
| Now | 5607(20.05) | 1131(15.40) | 1266(17.83) | 1462(21.61) | 1748(25.59) |  |
| Body Mass Index, n (%) |  |  |  |  |  | < 0.0001 |
| <25Kg/m^2^ | 7682(29.77) | 3006(46.91) | 2177(33.51) | 1510(23.56) | 989(14.51) |  |
| ≥25Kg/m^2^ | 19288(70.23) | 3738(53.09) | 4560(66.49) | 5239(76.44) | 5751(85.49) |  |
| Total energy intake (Kcal) Mean (S.E) | 2198.47(8.87) | 2084.80(15.15) | 2213.13(17.05) | 2247.24(15.98) | 2250.56(16.45) | < 0.0001 |
| Cancer, n (%) |  |  |  |  |  | < 0.0001 |
| No | 24439(90.16) | 5939(87.57) | 6083(89.65) | 6177(91.38) | 6240(92.09) |  |
| Yes | 2531(9.84) | 805(12.43) | 654(10.35) | 572(8.62) | 500(7.91) |  |
| Thyroid problem, n (%) |  |  |  |  |  | < 0.001 |
| No | 24205(89.11) | 5944(87.34) | 6031(88.91) | 6096(89.88) | 6134(90.36) |  |
| Yes | 2765(10.89) | 800(12.66) | 706(11.09) | 653(10.12) | 606(9.64) |  |
| Liver diseases, n (%) |  |  |  |  |  | 0.02 |
| No | 25931(96.55) | 6444(96.02) | 6509(97.34) | 6501(96.31) | 6477(96.52) |  |
| Yes | 1039(3.45) | 300(3.98) | 228(2.66) | 248(3.69) | 263(3.48) |  |
| Arthritis, n (%) |  |  |  |  |  | < 0.0001 |
| No | 19615(74.28) | 4695(71.77) | 4897(74.01) | 4970(75.74) | 5053(75.65) |  |
| Yes | 7355(25.72) | 2049(28.23) | 1840(25.99) | 1779(24.26) | 1687(24.35) |  |
| Diabetes, n (%) |  |  |  |  |  | < 0.0001 |
| No | 19666(77.82) | 5138(81.78) | 5040(79.96) | 4843(76.44) | 4645(72.90) |  |
| Yes | 7304(22.18) | 1606(18.22) | 1697(20.04) | 1906(23.56) | 2095(27.10) |  |
| CVD, n (%) |  |  |  |  |  | 0.1 |
| No | 24108(91.74) | 5950(90.99) | 6031(92.13) | 6065(91.68) | 6062(92.17) |  |
| Yes | 2862(8.26) | 794(9.01) | 706(7.87) | 684(8.32) | 678(7.83) |  |
| CKD, n (%) |  |  |  |  |  | < 0.0001 |
| No | 22226(86.08) | 5403(84.11) | 5618(86.89) | 5654(87.56) | 5551(85.76) |  |
| Yes | 4744(13.92) | 1341(15.89) | 1119(13.11) | 1095(12.44) | 1189(14.24) |  |
| Antidepressant utilization, n (%) |  |  |  |  |  | 0.42 |
| No | 24042(87.06) | 6053(86.99) | 6000(87.33) | 6037(87.61) | 5952(86.28) |  |
| Yes | 2928(12.94) | 691(13.01) | 737(12.67) | 712(12.39) | 788(13.72) |  |
| Antiplatelet utilization, n (%) |  |  |  |  |  | 0.8 |
| No | 26046(97.58) | 6528(97.75) | 6488(97.51) | 6508(97.54) | 6522(97.51) |  |
| Yes | 924(2.42) | 216(2.25) | 249(2.49) | 241(2.46) | 218(2.49) |  |
| Statins utilization, n (%) |  |  |  |  |  | < 0.0001 |
| No | 21875(83.21) | 5318(81.24) | 5389(82.43) | 5516(84.41) | 5652(84.84) |  |
| Yes | 5095(16.79) | 1426(18.76) | 1348(17.57) | 1233(15.59) | 1088(15.16) |  |

**Abbreviation:** HDL-C, high-density lipoprotein cholesterol; PHR, platelet-to-high-density lipoprotein cholesterol ratio; CVD, cardiovascular disease; CKD, chronic kidney disease; DM, diabetes; SE, standard error.

## Supplementary Table 4: Weighted characteristics of the eligible 2,102 participants in the analysis of PHR with CVD mortality among depression patients

| Characteristics | Total (N=2,102) | Alive (N=2,019) | CVD-specific death(N=83) | P value |
| --- | --- | --- | --- | --- |
| Follow-up time (years), Mean (S.E) | 7.57(0.16) | 7.65(0.17) | 5.06(0.50) | < 0.0001 |
| PHR, Mean (S.E) | 211.46(2.32) | 210.91(2.34) | 229.55(15.17) | 0.23 |
| Platelet (1000 cells/μL), Mean (S.E) | 259.15(1.96) | 258.94(1.94) | 266.17(11.61) | 0.53 |
| HDL-C (mmol/L), Mean (S.E) | 1.33(0.01) | 1.33(0.01) | 1.28(0.05) | 0.35 |
| Sex, n (%) |  |  |  | 0.75 |
| Female | 1353(63.83) | 1306(63.88) | 47(62.09) |  |
| Male | 749(36.17) | 713(36.12) | 36(37.91) |  |
| Age, n (%) |  |  |  | < 0.0001 |
| <40 years | 718(37.44) | 717(38.49) | 1(3.14) |  |
| 40−60 years | 878(44.12) | 853(44.44) | 25(33.91) |  |
| ≥60 years | 506(18.44) | 449(17.07) | 57(62.95) |  |
| Race, n (%) |  |  |  | 0.21 |
| Mexican American | 320(7.79) | 315(7.94) | 5(2.94) |  |
| Non-Hispanic Black | 436(12.46) | 418(12.46) | 18(12.28) |  |
| Non-Hispanic White | 922(65.25) | 872(64.93) | 50(75.62) |  |
| Other Hispanic | 269(7.34) | 261(7.47) | 8(3.21) |  |
| Others | 155(7.17) | 153(7.21) | 2(5.94) |  |
| Education attainment, n (%) |  |  |  | 0.004 |
| Less than college | 1205(50.53) | 1148(49.92) | 57(70.35) |  |
| College or higher | 897(49.47) | 871(50.08) | 26(29.65) |  |
| Marital status, n (%) |  |  |  | < 0.0001 |
| Never married | 452(21.54) | 447(22.12) | 5(2.59) |  |
| Divorced/separated/widowed | 693(29.66) | 644(28.76) | 49(58.95) |  |
| Married/living with a partner | 957(48.80) | 928(49.12) | 29(38.45) |  |
| Poverty, n (%) |  |  |  | 0.23 |
| <1.3 | 1081(39.55) | 1030(39.25) | 51(49.50) |  |
| 1.3–3.5 | 701(36.20) | 678(36.23) | 23(35.24) |  |
| >3.5 | 320(24.25) | 311(24.52) | 9(15.26) |  |
| Alcohol status, n (%) |  |  |  | < 0.0001 |
| Never | 248(8.70) | 235(8.27) | 13(22.87) |  |
| Former | 423(18.16) | 384(17.48) | 39(40.01) |  |
| Now | 1431(73.14) | 1400(74.25) | 31(37.12) |  |
| Smoke, n (%) |  |  |  | 0.89 |
| Never | 849(38.79) | 818(38.86) | 31(36.60) |  |
| Former | 465(22.43) | 447(22.45) | 18(21.80) |  |
| Now | 788(38.78) | 754(38.69) | 34(41.60) |  |
| Body Mass Index, n (%) |  |  |  | 0.09 |
| <25Kg/m^2^ | 496(25.76) | 468(25.45) | 28(35.75) |  |
| ≥25Kg/m^2^ | 1606(74.24) | 1551(74.55) | 55(64.25) |  |
| Total energy intake (Kcal) Mean (S.E) | 2090.77(29.86) | 2094.20(29.51) | 1979.13(150.28) | 0.44 |
| Cancer, n (%) |  |  |  | 0.11 |
| No | 1901(89.56) | 1830(89.75) | 71(83.13) |  |
| Yes | 201(10.44) | 189(10.25) | 12(16.87) |  |
| Thyroid problem, n (%) |  |  |  | 0.81 |
| No | 1773(83.69) | 1703(83.73) | 70(82.63) |  |
| Yes | 329(16.31) | 316(16.27) | 13(17.37) |  |
| Liver diseases, n (%) |  |  |  | 0.26 |
| No | 1937(92.96) | 1858(92.87) | 79(95.87) |  |
| Yes | 165(7.04) | 161(7.13) | 4(4.13) |  |
| Arthritis, n (%) |  |  |  | < 0.001 |
| No | 1203(59.18) | 1174(59.91) | 29(35.41) |  |
| Yes | 899(40.82) | 845(40.09) | 54(64.59) |  |
| Diabetes, n (%) |  |  |  | < 0.0001 |
| No | 1439(73.88) | 1413(75.00) | 26(37.30) |  |
| Yes | 663(26.12) | 606(25.00) | 57(62.70) |  |
| CVD, n (%) |  |  |  | < 0.0001 |
| No | 1736(85.84) | 1692(86.82) | 44(53.84) |  |
| Yes | 366(14.16) | 327(13.18) | 39(46.16) |  |
| CKD, n (%) |  |  |  | < 0.0001 |
| No | 1715(85.69) | 1682(86.89) | 33(46.75) |  |
| Yes | 387(14.31) | 337(13.11) | 50(53.25) |  |
| Antidepressant utilization, n (%) |  |  |  | 0.77 |
| No | 1430(63.40) | 1373(63.34) | 57(65.25) |  |
| Yes | 672(36.60) | 646(36.66) | 26(34.75) |  |
| Antiplatelet utilization, n (%) |  |  |  | < 0.0001 |
| No | 1992(95.56) | 1925(95.92) | 67(83.80) |  |
| Yes | 110(4.44) | 94(4.08) | 16(16.20) |  |
| Statins utilization, n (%) |  |  |  | < 0.001 |
| No | 1696(81.73) | 1642(82.34) | 54(62.03) |  |
| Yes | 406(18.27) | 377(17.66) | 29(37.97) |  |

**Abbreviation:** HDL-C, high-density lipoprotein cholesterol; PHR, platelet-to-high-density lipoprotein cholesterol ratio; CVD, cardiovascular disease; CKD, chronic kidney disease; DM, diabetes; SE, standard error.

## Supplementary Table 5: Sensitivity analysis of PHR with the risk of depression

| **Unweighted** |  | Model 0 | | Model 1 | | Model 2 | | Model 3 | |
| --- | --- | --- | --- | --- | --- | --- | --- | --- | --- |
|  |  | OR (95% CI) | P | OR (95% CI) | P | OR (95% CI) | P | OR (95% CI) | P |
|  | Quartile 1 | ref |  | ref |  | ref |  | ref |  |
|  | Quartile 2 | 1.22(1.08,1.39) | 0.002 | 1.23(1.08,1.40) | 0.002 | 1.17(1.02,1.33) | 0.03 | 1.17(1.02,1.35) | 0.02 |
|  | Quartile 3 | 1.36(1.20,1.55) | <0.0001 | 1.34(1.18,1.53) | <0.0001 | 1.19(1.04,1.36) | 0.01 | 1.20(1.05,1.38) | 0.01 |
|  | Quartile 4 | 1.68(1.49,1.90) | <0.0001 | 1.61(1.42,1.83) | <0.0001 | 1.33(1.17,1.52) | <0.0001 | 1.29(1.12,1.48) | <0.001 |
|  | p for trend |  | <0.0001 |  | <0.0001 |  | <0.0001 |  | <0.001 |
|  | per SD^+^ | 1.19(1.14,1.23) | <0.0001 | 1.17(1.13,1.22) | <0.0001 | 1.10(1.05,1.14) | <0.0001 | 1.07(1.03,1.12) | 0.001 |
|  |  |  |  |  |  |  |  |  |  |
| **Multiple interpolation for missing covariates** |  | Model 0 | | Model 1 | | Model 2 | | Model 3 | |
|  |  | OR (95% CI) | P | OR (95% CI) | P | OR (95% CI) | P | OR (95% CI) | P |
|  | Quartile 1 | ref |  | ref |  | ref |  | ref |  |
|  | Quartile 2 | 1.22(1.08,1.37) | <0.001 | 1.23(1.09,1.38) | <0.001 | 1.19(1.05,1.34) | 0.01 | 1.18(1.02,1.35) | 0.02 |
|  | Quartile 3 | 1.40(1.25,1.57) | <0.0001 | 1.40(1.25,1.57) | <0.0001 | 1.26(1.12,1.42) | <0.001 | 1.20(1.05,1.38) | 0.01 |
|  | Quartile 4 | 1.64(1.47,1.83) | <0.0001 | 1.62(1.44,1.81) | <0.0001 | 1.34(1.19,1.51) | <0.0001 | 1.29(1.12,1.48) | <0.001 |
|  | p for trend |  | <0.0001 |  | <0.0001 |  | <0.0001 |  | <0.001 |
|  | per SD^+^ | 1.18(1.14,1.22) | <0.0001 | 1.17(1.13,1.21) | <0.0001 | 1.10(1.06,1.14) | <0.0001 | 1.07(1.03,1.12) | 0.002 |
|  |  |  |  |  |  |  |  |  |  |
| **Additional adjustments to metS in model 3** |  | Model 0 | | Model 1 | | Model 2 | | Model 3 | |
|  |  | OR (95% CI) | P | OR (95% CI) | P | OR (95% CI) | P | OR (95% CI) | P |
|  | Quartile 1 | ref |  | ref |  | ref |  | ref |  |
|  | Quartile 2 | 1.25(1.04,1.50) | 0.02 | 1.27(1.06,1.52) | 0.01 | 1.17(0.98,1.41) | 0.09 | 1.20(0.99,1.46) | 0.06 |
|  | Quartile 3 | 1.43(1.19,1.70) | <0.001 | 1.42(1.19,1.71) | <0.001 | 1.24(1.03,1.48) | 0.02 | 1.24(1.02,1.50) | 0.03 |
|  | Quartile 4 | 1.71(1.46,2.02) | <0.0001 | 1.67(1.41,1.98) | <0.0001 | 1.34(1.12,1.60) | 0.002 | 1.31(1.09,1.57) | 0.004 |
|  | p for trend |  | <0.0001 |  | <0.0001 |  | 0.003 |  | 0.01 |
|  | per SD^+^ | 1.20(1.14,1.25) | <0.0001 | 1.18(1.13,1.24) | <0.0001 | 1.09(1.03,1.15) | 0.002 | 1.06(1.00,1.12) | 0.04 |

**Notes:**

Model 0: Not adjusted;

Model 1: Adjusted for age, gender, education attainment, and race;

Model 2: Further adjusted for marital status, poverty-income ratio, smoking and drinking status, BMI and total energy intake based on Model 1;

Model 3: Further adjusted for arthritis, thyroid problems, cancer, diabetes, liver diseases, CVD, CKD, Statins, and antiplatelets based on Model 2.

**Abbreviations**: PHR, platelet-to-high-density lipoprotein cholesterol ratio; metS, metabolic syndrome; BMI, body mass index; CVD, cardiovascular disease; CKD, chronic kidney disease; SD, standard deviation; OR, odds ratio; CI, confidence interval; ref, reference.

## Supplementary Table 6: Sensitivity analysis of PHR with the risk of CVD mortality among depression patients

| **Exclusion of cases that died within two years of follow-up** |  | Model 0 | | Model 1 | |
| --- | --- | --- | --- | --- | --- |
|  |  | HR (95% CI) | P | HR (95% CI) | P |
|  | per SD^+^ | 1.15(0.85,1.54) | 0.36 | 1.38(1.05, 1.81) | 0.02 |
|  | per 100^+^ | 1.17(0.84,1.63) | 0.36 | 1.44(1.06, 1.95) | 0.02 |

**Notes:**

Model 0: Not adjusted;

Model 1: Adjusted for age, race, BMI, DM, CVD, CKD, Statins, and antiplatelets.

**Abbreviation:** PHR, platelet-to-high-density lipoprotein cholesterol ratio; CVD, cardiovascular disease; BMI, body mass index; DM, diabetes; CKD, chronic kidney disease; SD, standard deviation; HR, hazard ratio; CI, confidence interval.

## Supplementary Table 7: Sensitivity analysis of per-SD increase in PHR with the risk of depression (Redefining Depression)

|  | Model 0 | | Model 1 | | Model 2 | | Model 3 | |
| --- | --- | --- | --- | --- | --- | --- | --- | --- |
|  | OR (95% CI) | P | OR (95% CI) | P | OR (95% CI) | P | OR (95% CI) | P |
|  | ref |  | ref |  | ref |  | ref |  |
| Additional analysis 1 | 1.10(1.06,1.15) | <0.0001 | 1.18(1.13,1.23) | <0.0001 | 1.10(1.05,1.15) | <0.0001 | 1.09(1.04,1.14) | <0.001 |
| Additional analysis 2 | 1.20(1.14,1.26) | <0.0001 | 1.16(1.10,1.22) | <0.0001 | 1.10(1.04,1.17) | 0.001 | 1.10(1.03,1.16) | 0.002 |
| Additional analysis 3 | 1.01(0.96,1.08) | 0.64 | 1.15(1.08,1.21) | <0.0001 | 1.09(1.03,1.16) | 0.002 | 1.09(1.03,1.15) | 0.01 |
| Additional analysis 4 | 1.21(1.15,1.28) | <0.0001 | 1.18(1.12,1.25) | <0.0001 | 1.12(1.05,1.19) | <0.001 | 1.12(1.05,1.19) | <0.001 |

**Notes:**

Additional analysis 1: Using antidepressants or PHQ-9 >= 10 was defined as depression in order to define some potential depressive populations as depression.

Additional analysis 2: Not using antidepressants and having PHQ-9 >= 10 was defined as depression (untreated depression).

Additional analysis 3: After excluding the participants with PHQ-9 >= 10 score, the individuals who use antidepressants and have PHQ-9 < 10 was defined as depression in order to explore the association between PHQ-9 and the effectively treated depression.

Additional analysis 4: Exclude the people who use antidepressants to rule out the interference of antidepressants.

Model 0: Not adjusted;

Model 1: Adjusted for age, gender, education attainment, and race;

Model 2: Further adjusted for marital status, poverty-income ratio, smoking and drinking status, BMI and total energy intake based on Model 1;

Model 3: Further adjusted for arthritis, thyroid problems, cancer, diabetes, liver diseases, CVD, CKD, Statins, and antiplatelets based on Model 2.

**Abbreviations**: PHR, platelet-to-high-density lipoprotein cholesterol ratio; BMI, body mass index; CVD, cardiovascular disease; CKD, chronic kidney disease; PHQ-9, Patient Health Questionnaire-9; SD, standard deviation; OR, odds ratio; CI, confidence interval.
